# Transcriptomic profiling of purple broccoli reveals light-induced anthocyanin biosynthetic signaling and structural genes

**DOI:** 10.7717/peerj.8870

**Published:** 2020-05-05

**Authors:** Chunqing Liu, Xueqin Yao, Guangqing Li, Lei Huang, Zhujie Xie

**Affiliations:** 1 Shanghai Academy of Agricultural Sciences, Institute of Horticulture, Shanghai, China; 2 School of Ecological Technology and Engineering, Shanghai Institute of Technology, Shanghai, China

**Keywords:** Broccoli, Transcriptome, Anthocyanins, Light induction

## Abstract

Purple Broccoli (*Brassica oleracea* L. var *italica*) attracts growing attention as a functional food. Its purple coloration is due to high anthocyanin amounts. Light represents a critical parameter affecting anthocyanins biosynthesis. In this study, ‘Purple Broccoli’, a light-responding pigmentation cultivar, was assessed for exploring the mechanism underlying light-induced anthocyanin biosynthesis by RNA-Seq. Cyanidin, delphinidin and malvidin derivatives were detected in broccoli head samples. Shading assays and RNA-seq analysis identified the flower head as more critical organ compared with leaves. Anthocyanin levels were assessed at 0, 7 and 11 days, respectively, with further valuation by RNA-seq under head-shading and light conditions. RNA sequences were de novo assembled into 50,329 unigenes, of which 38,701 were annotated against four public protein databases. Cluster analysis demonstrated that anthocyanin/phenylpropanoid biosynthesis, photosynthesis, and flavonoid biosynthesis in cluster 8 were the main metabolic pathways regulated by light and had showed associations with flower head growth. A total of 2,400 unigenes showed differential expression between the light and head-shading groups in cluster 8, including 650 co-expressed, 373 specifically expressed under shading conditions and 1,377 specifically expressed under normal light. Digital gene expression (DGE) analysis demonstrated that light perception and the signal transducers *CRY3* and *HY5* may control anthocyanin accumulation. Following shading, 15 structural genes involved in anthocyanin biosynthesis were downregulated, including *PAL*, *C4H*, 4CL, *CHS*, *CHI*, *F3H* and *DFR*. Moreover, six BoMYB genes (*BoMYB6-1*, *BoMYB6-2*, *BoMYB6-3*,* BoMYB6-4*, *BoMYBL2-1* and *BoMYBL2-2*) and three BobHLH genes (*BoTT8_5-1*, *BoTT8_5-2* and *BoEGL5-3*) were critical transcription factors controlling anthocyanin accumulation under light conditions. Based on these data, a light-associated anthocyanin biosynthesis pathway in Broccoli was proposed. This information could help improve broccoli properties, providing novel insights into the molecular mechanisms underpinning light-associated anthocyanin production in purple vegetables.

## Introduction

Broccoli (*Brassica oleracea* L. var. *italica*) represents a nutritious plant whose flower heads are rich in vitamins A, B2 and C, minerals, and antioxidant phytochemicals such as glucoraphanin (Moreno et al., 2010). Purple broccoli serves as a functional food with significant anthocyanin production in the head, leaves and seeds. In *Brassica* species, purple/red color conferred by anthocyanin accumulation is tightly associated with the induction of structural genes and transcription factors (Song et al., 2018). A previous study showed that insertion of Harbinger transposon of *BoMYB2* results in its upregulation, which then together with BobHLHs upregulates the downstream genes of anthocyanin biosynthesis in purple cauliflower (Chiu et al., 2010). In red cabbage, *TT8* and *MYB2* upregulate structural genes in the anthocyanin pathway (Yuan, Chiu & Li, 2009) as well as the structural genes *F3’H*, *DFR* and *ANS* in kale (Zhang et al., 2012; Neugart, Krumbein & Zrenner, 2016). In addition, deletion or substitution in the coding sequences of *BoMYBL2-1* leads to purple color accumulation of cabbage (Song et al., 2018). In Kohlrabi, *BoPAP1* promotes purple coloration in a light dependent manner as well as *BoCHS* while *BoTT8* and *BoPAP2* are light-independent (Zhang et al., 2015). Some candidate genes controlling color formation have also been mapped in Brassica crops. In broccoli, a major locus (*qPH*.*C01*-*2*) and two minor loci (*qPH*.*C01*-*4* and *qPH*.*C01*-5) for purple sepal trait of the flower head were mapped onto chromosome C01 (Yu et al., 2019). In ornamental kale, single genes for pink or purple leaf traits were mapped onto chromosome C03 ( Zhu et al., 2016) and C09 (Liu et al., 2017), respectively. The change in color of inner and outer flower head (from green to purple) in broccoli is induced by light. However, the mechanism underlying purple color formation remains largely undefined in broccoli.

Anthocyanins comprise the largest subclass of hydrosoluble pigments conferring red, orange, purple, and blue colorations to flowers, fruits, seeds, and vegetables in plants (Cominelli et al., 2008; Espley et al., 2007; Li et al., 2017). Anthocyanins play important roles in furnishing flowers and fruits for attracting pollinations and dispersers, protecting plants from various biotic and abiotic stressors (Harborne & Williams, 2000; Ahmed et al., 2014). In addition, the health benefits of anthocyanins attract growing attention due to their antioxidant activities (Pojer et al., 2013). Genetic parameters, developmental stages and environmental conditions control anthocyanin accumulation, including high-light, UV-light, cold temperature, nutrient availability and infection (Dixon & Paiva, 1995; Chalker-Scott, 1999). Based on the above, a comprehensive understanding of regulation of anthocyanin biosynthesis should be investigated to obtain anthocyanin-rich foods via breeding and/or environmental control.

Anthocyanin biosynthesis has been assessed in various species such as *Arabidopsis*, petunia, tobacco, and fruit plants (Liu, Osbourn & Ma, 2015). The genes of the anthocyanin biosynthetic pathway are well-conserved across species (Holton & Cornish, 1995; Zhang, Butelli & Martin, 2014; Guo et al., 2014). Anthocyanin synthesis starts with phenylalanine, and is catalyzed stepwise by phenylalanine ammonia lyase (PAL), 4-coumarate-CoA ligase (4CL), chalcone synthase (CHS), chalcone isomerase (CHI), flavanone 3-hydroxyl enzyme (F3H), flavonoid 3′-hydroxylase (F3’H), dihydroflavonol reductase (DFR) and anthocyanin synthase (ANS) or leucoanthocyanidin dioxygenase (LDOX).

These structural genes are transcriptionally controlled by the MBW complex composed of R2R3-MYB, basic Helix-Loop-Helix (bHLH) and WD repeat protein (WDR). In Eudicots, R2R3-MYB transcription factors (TFs) are primarily implicated as positive regulators to initiate and activate the MBW complex in leaves under stress and young flowers/fruits. It was reported for the first time in maize that R2R3-MYB TFs C1 regulates anthocyanin synthesis (Paz-Ares et al., 1986), and co-action of bHLH proteins and Leaf color (Lc) was found recently (Ludwig et al., 1989). *AtMYB75*/*PAP1*, *AtMYB90*/*PAP2*, *AtMYB112, AtMYB113* and *AtMYB114* contribute to anthocyanin production in *Arabidopsis* (Gonzalez et al., 2008; Lotkowska et al., 2015). In petunia, *AN1* and *AN2* contribute to anthocyanin synthesis and vacuolar acidification (Koes, Verweij & Quattrocchio, 2005); meanwhile, *PyMYB10* and *PyMYB10.1* bind to bHLH for enhancing anthocyanin production in pears (Feng et al., 2015). However, some MYB TFs, such as *AtMYB3*/*4*/*6*/*60* (Jin et al., 2000) and *AtMYBL2* (Matsui, Umemura & Ohme-Takagi, 2008), *FaMYB1* and *FcMYB1* (Aharoni et al., 2001; Zhang et al., 2018), *VvMYB4*/*C2* (Matus, Aquea & Arce-Johnson, 2008), and *MdMYB16*/*17*/*111* (Lin-Wang et al., 2011), exert inhibitory effects. These negative regulators interact with the bHLH protein, thereby competing with R2R3-MYB activators. The bHLH proteins controlling anthocyanin production have been described in multiple species, including *Arabidopsis* (*GL3*, *EGL3* and *TT8*) (Payne, Zhang & Lloyd, 2000; Zhang et al., 2003; Nesi et al., 2000), petunia (*PhAN1*, *PhJAF13*) (Spelt et al., 2000; Quattrocchio et al., 1998), apple (*MdbHLH3*/*MdbHLH33*) (Espley et al., 2007), grape (*VvMYC1*) (Hichri et al., 2010) and peach (*PpbHLH3*/*PpbHLH33*) (Ravaglia et al., 2013). The various transcriptional modulators control anthocyanin production as well as their modifications and translocation into vacuoles via glutathione S-transferases (GSTs), the ATP-binding cassette (ABC) and multidrug and toxic compound extrusion (MATE) proteins ( Wang et al., 2017).

Multiple parameters including genetic, developmental and environmental factors control anthocyanin biosynthesis. Light indexes, including intensity and quality, represent critical factors affecting anthocyanin accumulation (Albert et al., 2009). In lettuce and turnip, UV-A and UV-B increase anthocyanin contents via upregulation of *DFR* and *CHS* in the anthocyanin biosynthetic pathway (Zhou et al., 2007; Park et al., 2007). In the presence of light, photoreceptors are activated, including PHYs (*PHYA* to *E*) that absorb red/far-red light; CRYs (*CRY1* to *3*) and PHOTs (*PHOT1* and *2*) that sense blue/UV-A light, and *UVR8* that absorbs UV-B (Zoratti et al., 2014), interacting with the ubiquitin E3 ligase *COP1* (*CONSTITUTIVE PHOTOMORPHOGENIC1*) that controls the degradation of target transcription factors, including *ELONGATED HYPOCOTYL5* (*HY5*). *HY5* is associated with induced *CHS*, *CHI* and flavonoid production under light and UV-B radiation conditions in *Arabidopsis* (Shin, Park & Choi, 2007). It also binds the *MYB75*/*12*/*111* promoter to increase their expression and modulate anthocyanin biosynthesis (Stracke et al., 2010; Shin et al., 2013; Nguyen et al., 2015).

The present work aimed to assess the global transcription of regulatory, structural and hormone signal transduction genes which might positively or negatively regulate broccoli’s anthocyanin biosynthetic pathway. The light sensitivity of pigment biosynthesis makes the broccoli ‘Long Jing’ an optimal plant for evaluating anthocyanin synthesis and explore the underpinning mechanisms under light conditions. Indeed, anthocyanin accumulation accompanied with broccoli head growth and development under natural light conditions. However, broccoli heads show reduced coloration under shading conditions. Here, the ‘Long Jing’ cultivar was employed for analyzing light-associated anthocyanin biosynthesis and the underlying mechanisms by RNA-seq. Based on the association of shading time with anthocyanin amounts, anthocyanin accumulation was assessed at 0 d, 7 d (highest production rate) and 11 d (peak amounts). The present findings provide insights into the molecular mechanisms underpinning light-associated anthocyanin production in broccoli, facilitating genetic engineering for increasing anthocyanin amounts in vegetables.

## Materials & Methods

### Plant materials and RNA preparation

The purple broccoli cultivar ‘Long Jing’ was assessed as the experimental material. After flower head formation, a total of seven stages were defined as 0, 3, 5, 7, 9, 11, and 14 days. Flower heads were collected for phenotype observation and anthocyanin level measurement at each developmental stage of the head under light and dark (shading using a sunshade net over the whole heads or leaves) conditions. In addition, light and darkness treated heads at 0, 7 and 11 days, and darkness treated leaves at 7 days were collected for RNA-Seq from the flower heads of the purple cultivar, respectively. Three biological replicate specimens were obtained. All specimens underwent snap freezing in liquid nitrogen and storage at −80 °C. To assess cells displaying purple coloration, head flowers underwent transverse sectioning by hand and analysis under a Zeiss Axioscope photo microscope.

### Quantification of total anthocyanin amounts

Total anthocyanin amounts were determined by the pH differential method (Lee, Durst & Wrolstad, 2005) with some modifications. Briefly, 100 mg of fresh flowers were soaked in 500 µl acidified methanol (1% v/v formic acid), sonicated (15 min × 3 times), and centrifuged for 5 min at 4,000 rpm. The precipitate was extracted twice. All supernatants were collected and stored at −20 °C. Then, 200 µl samples were mixed with 800 µl KCl (pH 1) and 800 µl NaAc (pH 4.5), respectively. After incubation at room temperature for 20 min in the dark, optical density was read at 510 and 700 nm. The following equation was employed to derive anthocyanin amounts: total anthocyanin amounts (mg C3G/g FW) = A × MW × DF × V /(ε × 1 × m), where A = (A510 nm–A700 nm)pH 1.0–(A510 nm–A700 nm)pH 4.5; A510 and A700 are absorbance values at 510 and 700 nm, respectively; MW = 449.2; DF = 5; V is the total volume of supernatants; ε = 26900; m is fresh sample weight. Five replicates were assessed per biological specimen.

### Qualitative analysis of anthocyanin compounds by high- performance liquid chromatography (HPLC)

For HPLC, specimens underwent freeze-drying and pulverization in liquid nitrogen. Then, 100 mg of each specimen was thoroughly mixed for 5 min in 2 ml of 5% formic acid (v/v) in ultrapure water, and submitted to sonication (20 min). Upon centrifugation (12,000 rpm, 10 min), the supernatants underwent filtration with 0.45 µm PTFE filters (Toyo Roshi Kaisha, Japan). Anthocyanin amounts were assessed on an Agilent 1200 series HPLC (Agilent Technologies, USA), on a reverse phase Kromasil C18 column (5 µm; C18 80A, 250 × 4.60 mm; Kromasil) at 40 °C. Samples were injected at 10 µl, and a flow rate of 1 ml min^−1^ was adopted. Mobile phases A (1.6% formic acid) and B (methanol containing 0.01% formic acid) were employed as eluents: 0–5 min, 85% A+15% B; 5–10 min, 80% A+20% B; 10–20 min, 72% A+28% B; 20–35 min, 40% A+60% B; 35–35.1 min, 100% B; 35.1–50 min, 100% B; 50–50.1 min, 85% A+15% B; 50.1–60 min, 85% A+15% B. Detection was performed at 530 nm. Individual anthocyanins were quantitated (mg g-1 dry weight) via comparisons of areas under their HPLC peaks with those of known standards (delphinidin-3-O-galactoside, delphinidin-3-O-glucoside, cyanidin-3-O-galactoside, cyanidin-3-O-glucoside, malvidin-3-O-galactoside and malvidin-3-O-glucoside).

### RNA purification and library generation for transcriptomics

Total RNA was extracted from head flowers in purple broccoli at different times under both light conditions with mirVana miRNA Isolation Kit (Ambion) as directed by the manufacturer. RNA integrity was assessed on an Agilent 2100 Bioanalyzer (Agilent Technologies). Specimens with RNA Integrity Number (RIN) ≥7 were further evaluated. The libraries were generated with TruSeq Stranded mRNA LT Sample Prep Kit (Illumina, USA) as instructed by the manufacturer.

### Transcriptome sequencing, de novo assembly and functional annotation

The obtained libraries underwent sequencing by the PE strategy on a HiSeqTM 2500 or Illumina HiSeq X Ten; cDNA fragments approximated 125 or 150 bp. The raw reads obtained underwent pre-processing with Trimmomatic; those with ploy-N or showing low quality were excluded, leaving clean reads. These clean reads underwent assembly into contigs and *de novo* assembly into transcripts using Trinity (version: 2.4) (Grabherr et al., 2011) by the paired end method. The longest transcripts were selected as unigenes for further assessment. Original sequencing data were deposited in SRA (Short Read Archive; accession number PRJNA560282).

### Unigene quantification, assessment of differentially expressed genes (DEGs) and gene annotation

Fragments per kilobase of transcript per Million (FPKM) and read counts for each unigene were assessed with Bowtie 2 and eXpress. DEG identification was carried out with the DESeq functions estimate Size Factors and negative binomial Test. *P* < 0.05 and fold Change >2 or <0.5 were thresholds for determining significant differential expression. Hierarchical clustering of DEGs was carried out to assess the transcripts’ expression patterns. The assembled unigenes were assessed with R according to hypergeometric distribution.

### GO and KEGG pathway analyses of DEGs

The assembled unigenes underwent annotation by alignment in protein databases, including the NCBI non redundant (NR), SwissProt (http://www.expasy.ch/sprot), and Clusters of orthologous groups for eukaryotic complete genomes (COG) databases (https://www.ncbi.nlm.nih.gov/COG/) with Blastx (E<10^−5^). Proteins best matching the unigenes were employed for assigning functions. Based on the SwissProt annotation, GO classification was carried out using the Blast2GO software (Du et al., 2010; Conesa et al., 2005). Unigenes were mapped to the KEGG database (http://www.genome.jp/kegg) for determining the associated metabolic pathways (Kanehisa et al., 2008).

### Real-time quantitative reverse transcription-PCR

To confirm the results of Illumina analysis, qRT-PCR was performed for multiple genes. Total RNA isolation from specimens collected in various developmental stages under light or dark conditions was carried out. Reverse-transcription used PrimeScript RT Master Mix Perfect Real Time Kit (Takara) as directed by the manufacturer. Finally, qRT-PCR was carried out on a QuantStudio 5 Real-Time PCR System (Fisher Scientific, USA) with SYBR Premix Ex Taq (TaKaRa, Japan) in triplicate. Data were normalized to Actin 2 amounts, and the comparative CT method was employed for analysis.

## Results

### Phenotypic characterization of purple broccoli responding to light during anthocyanin production

**Figure 1:**
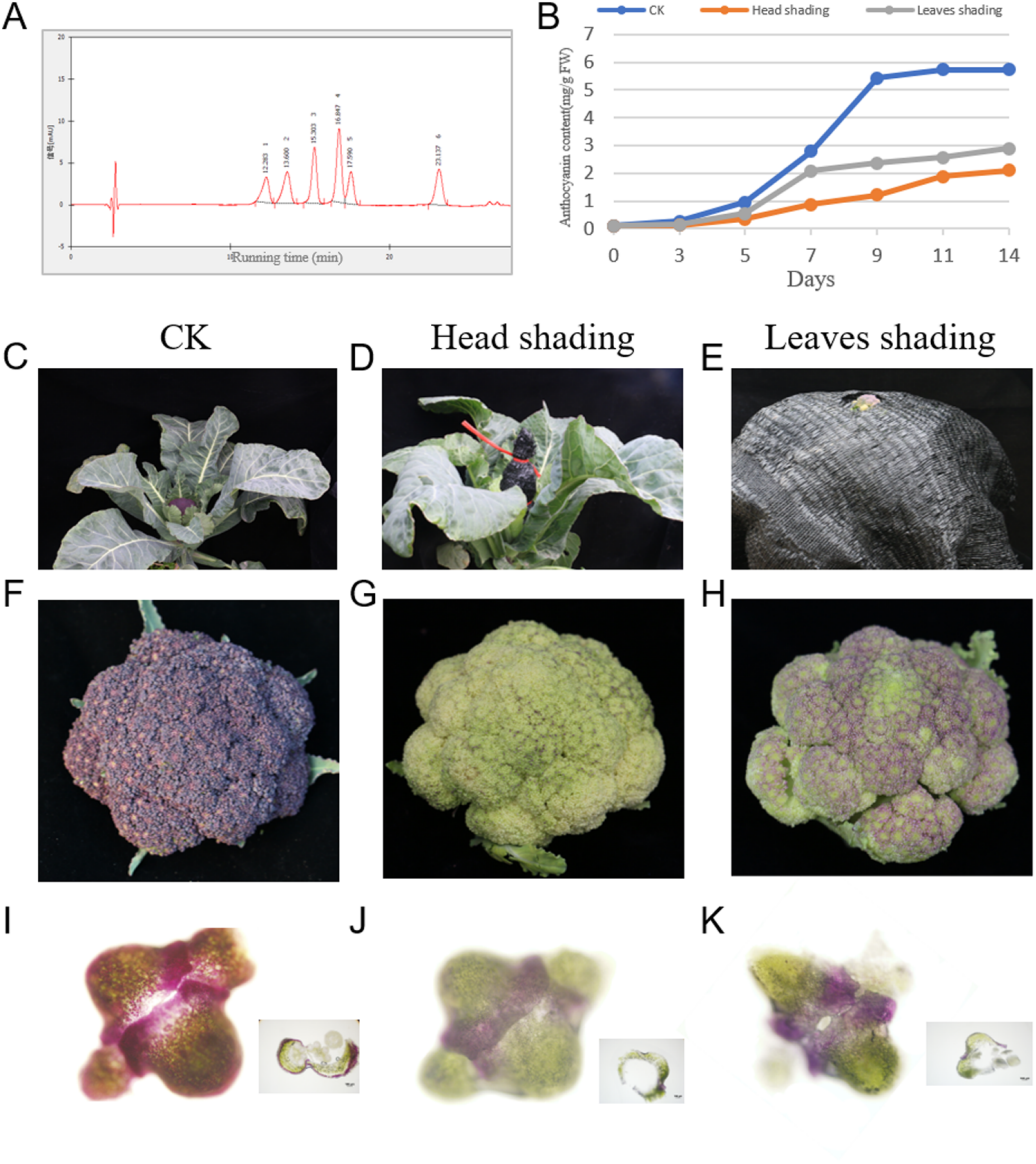
Accumulation of anthocyanins under head-shading, leaves-shading and normal light (CK) treatment in Broccoli. (A) Mass spectrometry assessment of anthocyanin levels by HPLC; (B) Relative anthocyanin amounts during head flower development; (C–E) Various shading treatments; (F–H) Respective head phenotypes under various treatments; (I–K) Anthocyanin levels in various parts of purple head flowers revealed by hand section.

The color of purple broccoli cultivar ‘Long Jing’ head is affected by pigment types and contents in flower buds as well as light during the developmental stages. Firstly, pigment types in broccoli heads were evaluated. Anthocyanin amounts were assessed by HPLC multistage tandem mass spectrometry (Fig. 1A). Peaks 1, 2, 3, 4, 5 and 6 were identified as delphinidin-3-O-galactoside, delphinidin-3-O-glucoside, cyanidin-3-O-galactoside, cyanidin-3-O-glucoside, malvidin-3-O-galactoside and malvidin-3-O-glucoside, respectively.

To study light-response factors in anthocyanin production in the broccoli cultivar ‘Long Jing’, we shaded the whole head and leaves using a sunshade net during the developmental stages of the head from 0 d to 14 d, with light conditions employed as a control treatment. In comparison with controls, flower buds grown under head- and leaves-shading all faded, with head shading exerting more pronounced effects (Fig. 1C). The relative amounts of total anthocyanin were measured, and the head-shading treatment group showed lower levels compared with the leaf-shading treatment group, and both of these groups had lower values than the control group (Fig. 1B; Table S1). In addition, decrease in relative anthocyanin levels was more pronounced under head-shading (2.04 mg g^−1^ FW) compared with leaf-shading (1.47 mg g^−1^FW), indicating that shading during the head development in broccoli significantly affected the relative contents of total anthocyanin.

Quantitative analysis was further conducted to identify the key developmental stage under light during anthocyanin production. Spectra were obtained at 200–600 nm, and chromatograms at 520 nm (Fig. 1B). In the control group, relative anthocyanin amounts rose during head development (0 d to 11 d), showed a peak growth rate at 7 d, and then remained steady after 11 d, while in shaded plants they increased slowly during head’s developmental stages from 0 d to 14 d. Therefore, 0 d, 7 d and 11 d were considered critical times for response to light during anthocyanin production.

Microscopic examination of sections of flower buds from head shading, leaf shading and control plants revealed that the prominent purple color extended to more bud tissue cells, with anthocyanins accumulating closer to the outer layer of buds (Fig. 1D), in accordance with previously published data on purple Graffiti cauliflower (Chiu & Li, 2012). Compared with the control treatment, head and leaves shading conditions resulted in lighter purple pigments in upper epidermal layers. The above results suggested light had a pivotal function in anthocyanin production, and shading treatment significantly repressed anthocyanin accumulation during head development in broccoli.

### Organ responses to light during anthocyanin biosynthesis

In order to assess differences in organ response to light during anthocyanin production in broccoli, RNA-Seq was performed under head-shading, leaves-shading and normal night conditions at the fastest point (7 d), respectively. The flowers were collected to construct nine libraries for transcriptomics in three biological replicates.

**Figure 2:**
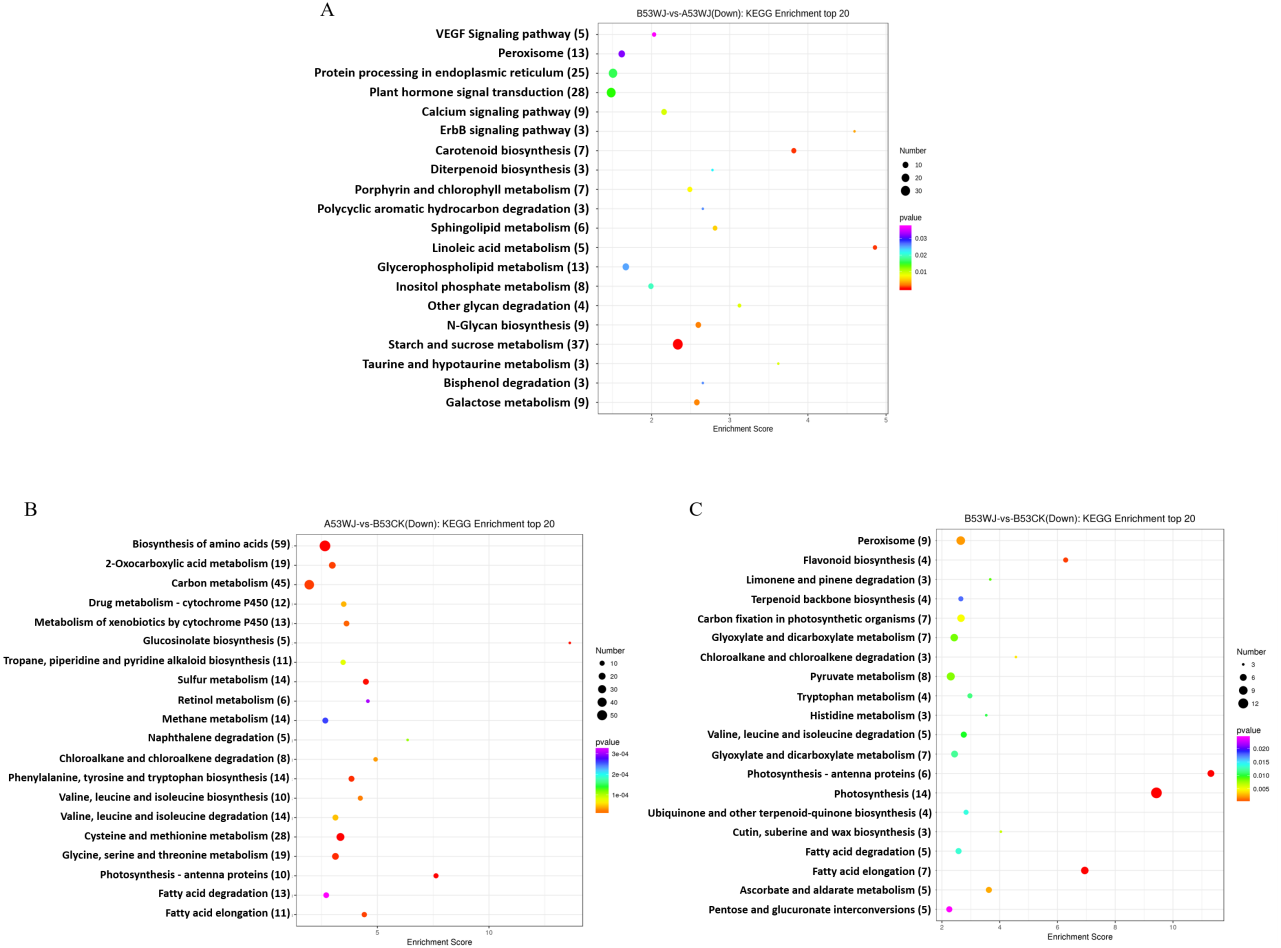
KEGG function classification of DEGs in B53WJ-vs-A53WJ (A), A53WJ-vs-B53CK (B) and B53WJ-vs-B53CK (C). B53WJ-vs-A53WJ refers to the number of DEGs at 7 d between head-shading and leaf-shading treatments; A53WJ -VS-B53CK refers to the number of DEGs at 7 d between leaf-shading and light treatments; B53WJ -VS-B53CK refers to the number of DEGs at 7 d between head-shading and light treatments.

Comparing the expression amounts of differently expressed genes (DEGs) under head-shading and leaves-shading treatment, a total of 2,223 and 2,558 DEGs were up- and down-regulated, respectively. To identify the functions of these down-regulated DEGs, Gene ontology (GO) and Kyoto Encyclopedia of Genes and Genomes (KEGG) analyses were carried out (Du et al., 2010; Conesa et al., 2005; Kanehisa et al., 2008). Thirty GO terms were found as enriched biological processes based on the DEGs (Table S2; Fig. S1): “nucleus”, “response to abscisic acid”, “DNA-binding transcription factor activity”, “response to water deprivation” and “sequence-specific DNA binding”, which were the major GO terms. The significantly enriched pathways were identified using KEGG analysis (Fig. 2A). Among the significantly enriched pathways, “Starch and sucrose metabolism”, “Plant hormone signal transduction” and “Protein processing in endoplasmic reticulum” were the major public pathway-related database.

Comparing the expression amounts of differently expressed genes (DEGs) under shading and normal light treatment, a total of 378 and 660 DEGs were up- and down-regulated under head-shading treatment, respectively. For DEGs under leaf-shading treatment, we found that a total of 1532 and 1628 DEGs were up- and down- regulated, respectively (Tables S3 and S4; Fig. S2 and S3). In GO analysis, DEGs encoding proteins related to response to “light stimulus”, “chloroplast and photosystem I” were down-regulated under both head- and leaf-shading treatments. In KEGG analysis, DEGs were grouped in 20 functional classes. Under leaf-shading treatment, DEGs were significantly enriched in “amino acid biosynthesis”, “carbon metabolism”, “sulfur metabolism”, “cysteine and methionine metabolism”, and “photosynthesis-antenna proteins” (Fig. 2B), while downregulated DEGs with previously described functions were associated with “photosynthesis”, “peroxisome” and “flavonoid biosynthesis”, indicating such pathways/processes might be affected by head shading (Fig. 2C). The KEGG analysis showed the critical pathways in response to light. The above results indicated that photosynthesis and anthocyanin biosynthetic process were markedly inhibited by head shading while photosynthesis and carbon-nitrogen-sulfur metabolism were significantly repressed by leaf shading by transcriptional regulation in response to light.

According to flower head phenotypes, pigment synthesis and associated DEGs, the head might constitute the main organ showing a response to light during anthocyanin biosynthesis, in accordance with previous data on chrysanthemum in which the capitulum was the key organ responding more to light compared with the leaf during anthocyanin production (Hong et al., 2015).

### Overall transcriptomic analysis under head shading and control treatments

**Figure 3:**
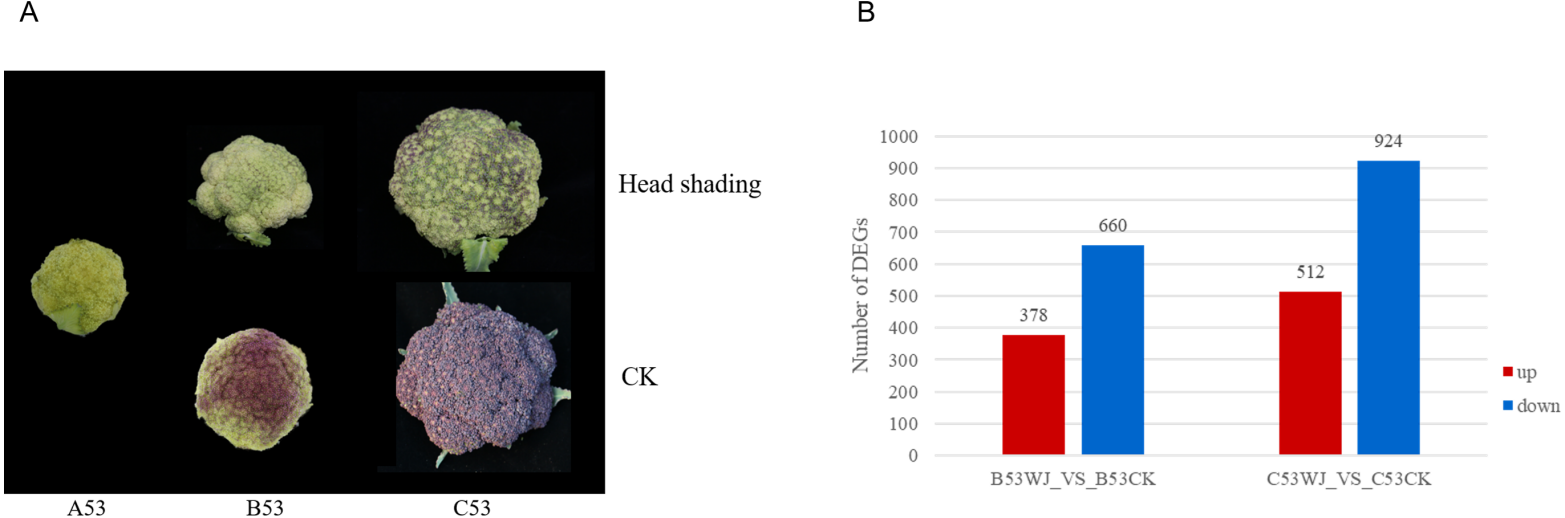
Differentially expressed genes (DEGs) in the broccoli head treated with head-shading and normal light conditions. (A) Head-shading treatment and corresponding head flower phenotypes during head flower development at 0 d (A53), 7 d (B53) and 11 d (C53); (B) Changes in gene expression at 7 d and 11 d. B53WJ -VS-B53CK refers to the number of DEGs at 7 d between the head-shading and light treatments. C53WJ-VS-C53CK refers to the amounts of DEGs at 11 d between the head-shading and light treatments.

To assess how light induces anthocyanin production in broccoli, RNA-Seq was performed for three triplicate groups at 0 d, 7 d and 11 d under head-shading and normal light treatments, respectively (Fig. 3A). Averagely 20 million clean reads were produced per sample, including 81∼85% which were mapped to the *Brassica oleracea* genome (Table S5). Cumulatively, 90231 transcripts were detected across all six treatments with an FPKM ≥1. The full annotation and the expression levels of all genes (FPKM values) are found in Tables S6 and S7.

### Thousands of genes are activated in broccoli in response to light

To identify genes responding to light, differential gene expression analyses were performed at 0 d, 7 d and 11 d under both head-shading and control treatments. Transcriptomics revealed 1461 (7 versus 0 day) and 5132 (11 versus 0 day) DEGs under control treatment, and 1752 (7 versus 0 day) and 1859 (11 vs. 0 day) DEGs under head-shading treatment. The number of shared DEGs increased over time, probably due to the total number of DEGs increasing between treatments. Relative to the control treatment, 378 upregulated and 660 downregulated DEGs (*P* < 0.05) were detected at 7 d, while 512 upregulated and 924 downregulated DEGs were found at 11 days, respectively (Fig. 3B).

**Figure 4:**
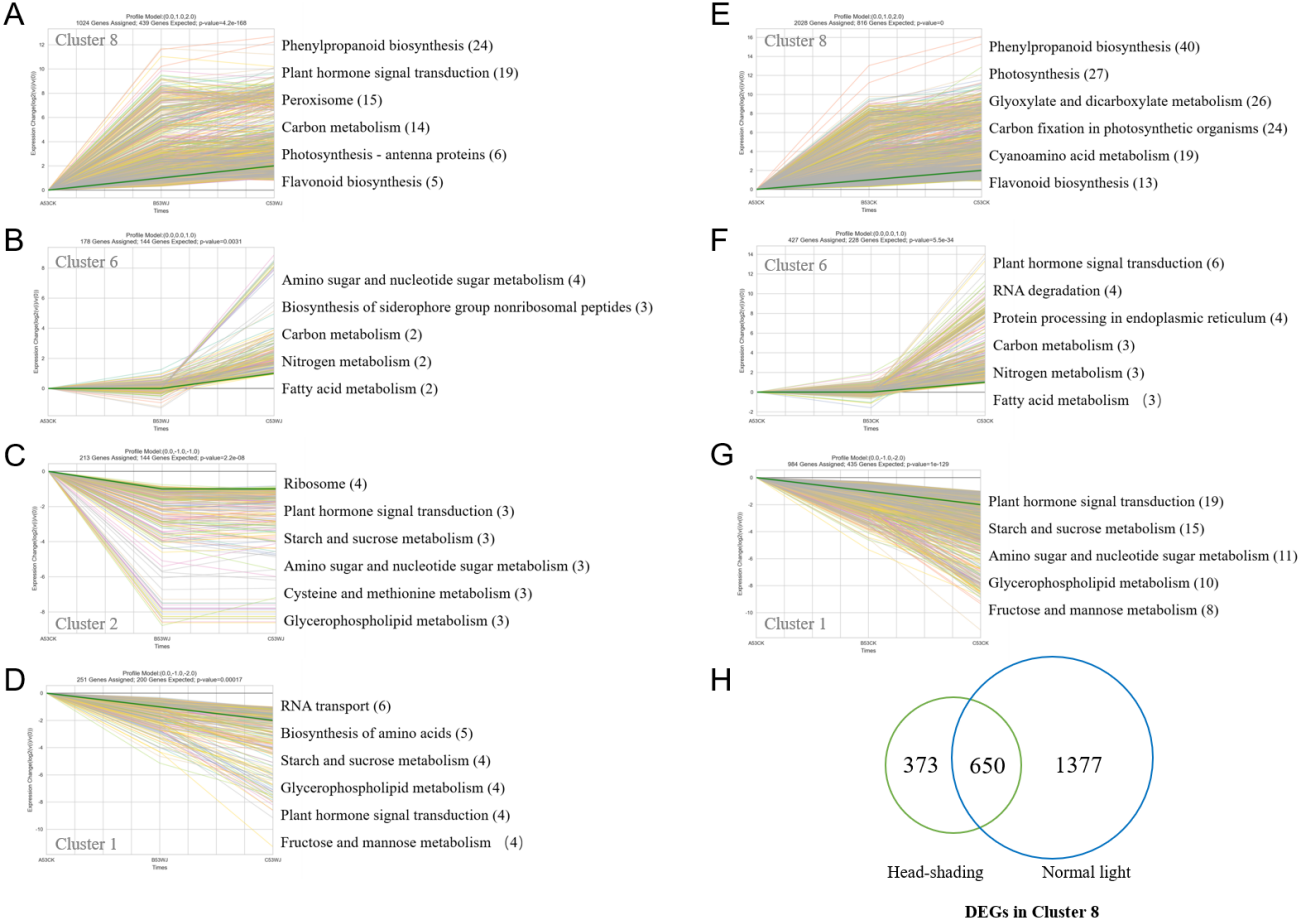
Clustering of differentially expressed genes with marked expression level differences and KEGG pathway analysis after head-shading treatment (A–D) and light treatment (E–G); (H) Overlap of DEGs in cluster 8 in response to light.

To gain a deeper understanding regarding the associated biological processes, transcripts were assigned to nine clusters per treatment group (Figs. S4 and S5). Then, KEGG analysis was performed for identifying biological pathways enriched in clusters of comparably regulated genes (Figs. 4A and 4B). Cluster 1, 6 and 8 were under both light and head-shading treatments. Cluster 1 encompassed genes downregulated throughout the study, including those controlling Plant hormone signal transduction, Starch and sucrose metabolism, Glycero-phospholipid metabolism and Fructose and mannose metabolism. Cluster 6 comprised genes positively regulated throughout the entire study, including those associated with Carbon metabolism, Nitrogen metabolism and Fatty acid metabolism. Cluster 8 contained genes significantly upregulated from 0 d to 7 d, many of which were involved in Phenylpropanoid biosynthesis, Photosynthesis, and Flavonoid biosynthesis. These findings indicated that cluster 8 DEGs were expressed in early phases of light induction and played roles in regulating anthocyanin biosynthesis. As many as 2400 DEGs showed differential expression between the light and shading libraries in cluster 8, in which 373 and 1377 individual DEGs showed specific expression under head-shading and normal light conditions, respectively, and 650 genes were co-expressed under both conditions (Fig. 4C; Table S8, S9 and S10). Thus, we focused on the 650 co-expressed and 373 specifically expressed under head-shading, which most likely represented light-induced genes.

### Different expression patterns of anthocyanin biosynthesis structural genes

**Figure 5:**
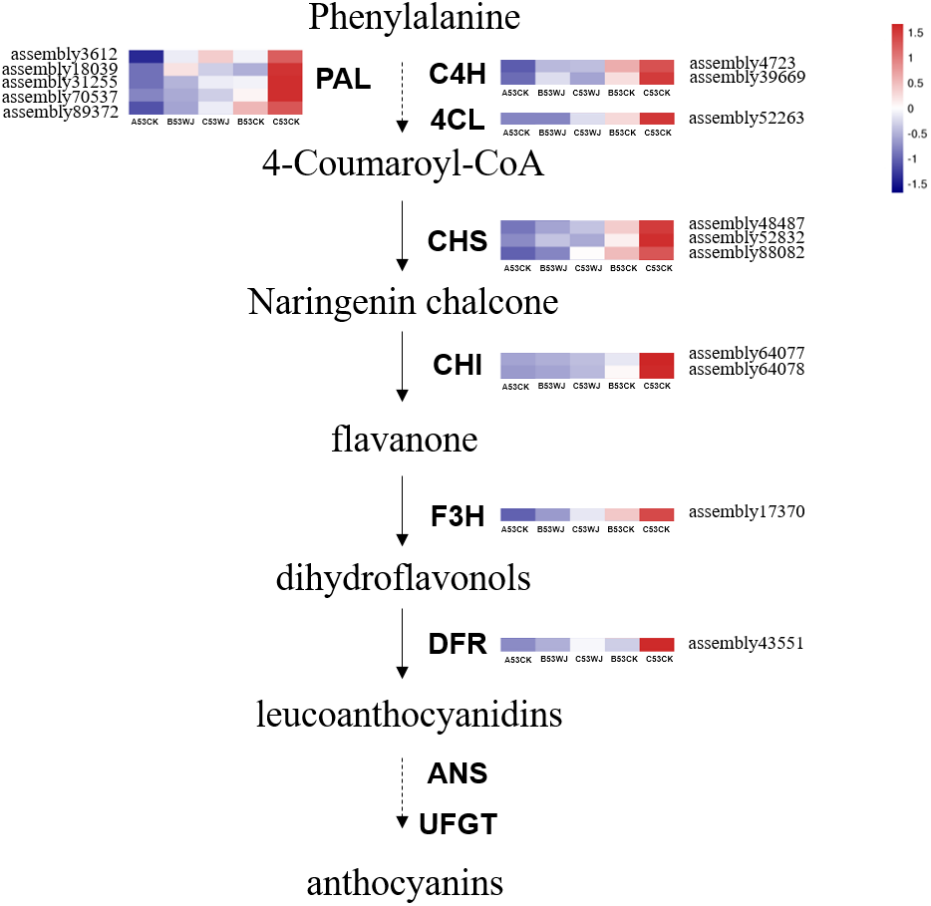
The flavonoid biosynthetic pathway associated with anthocyanin biosynthesis. The expression patterns of various structural genes contributing to anthocyanin production in A53CK, B53WJ, C53WJ, B53CK and C53CK are arranged from left to right. A53CK, B53WJ, C53WJ, B53CK and C53CK indicate samples treated by head-shading for 0 d, 7 d and 11 d, and normal light for 7 d and 11 d, respectively. The color scale reflected the log-transformed FPKM values.

The expression profiling of structural genes was performed to assess their roles in anthocyanin biosynthetic pathway after shading. In this study, fifteen such genes, e.g., *PAL*, *C4H*, *4CL*, *CHS*, *CHI*, *F3H* and *DFR* were regulated by light (Fig. 5). Most of the genes had comparable expression profiles; their expression amounts were low in the A53CK sample at 0 d, and gradually increased in the B53CK sample exposed to normal light for 7 d, peaking in the C53CK sample treated by normal light for 11 d. The expression levels of all transcripts were suppressed in the head-shading treatment group, corroborating the reduced anthocyanin amounts in plants under head-shading. Most genes showed higher expression levels in the C53WJ group treated by head-shading for 11 d compared with the B53WJ group treated by head-shading for 7 d, except assembly18039 (*PAL*), assembly 39669 (*C4H*) and assembly 52832 (*CHS*), which showed higher expression levels in the B53WJ group. Thus, these genes were considered critical structural genes associated with the effects of light on anthocyanin production.

### Expression of genes associated with light signal perception and transduction

Photoreceptors (PHYA, PHYB, PHYC, PHYD, PHYE), cryptochromes (CRY1, CRY2, CRY3), phototropins (PHOT1, PHOT2) and UV RESISTENCE LOCUS8 (UVR8) are four classes of photoreceptors contributing to light response (Chaves et al., 2011; Christie, 2007; Jenkins, 2014). Compared with head shading samples, flower head response to light was mediated by three *CRY3* photoreceptors, including assembly 37255, assembly 86925 and assembly 18166 (Table 1), which were all downregulated under head-shading treatment. Under light conditions, the expression levels of assembly 37255 and assembly 18166 gradually increased and showed highest values in C53CK under light treatment, while assembly 86925 showed the highest value in B53CK under light treatment.

**Table 1:** Genes expression related to photoreceptors and light signal transduction.

**Accession**	**Gene description (blast NR)**	**FPKM value**				
		A53CK	B53WJ	C53WJ	B53CK	C53CK
**photoreceptors**						
assembly37255	CRY3	0.16	0.41	0.21	3.05	5.01
assembly86925	CRY3	0.00	0.87	0.00	3.32	2.12
assembly18166	CRY3	0.00	0.57	0.17	2.18	3.48
**genes related to light signal transduction**						
assembly42644	HY5, TED 5	5.57	8.08	22.31	17.71	58.64

**Notes.**

CRY Cryptochrome HY5 HYPOCOTYL 5

*HY5* induces photomorphogenesis under all light conditions and exerts direct regulatory effects on light-responsive genes (Chattopadhyay et al., 1998). Here, we found *BoHY5* (assembly 42644) was downregulated under head-shading treatment in comparison with light conditions. Assembly 42644 showed rapid upregulation in the B53CK group, peaking in C53CK, with reduced amounts under head-shading treatment.

### DEGs Encoding Transcription Factors and their interaction with Hormone-Related Genes

**Figure 6:**
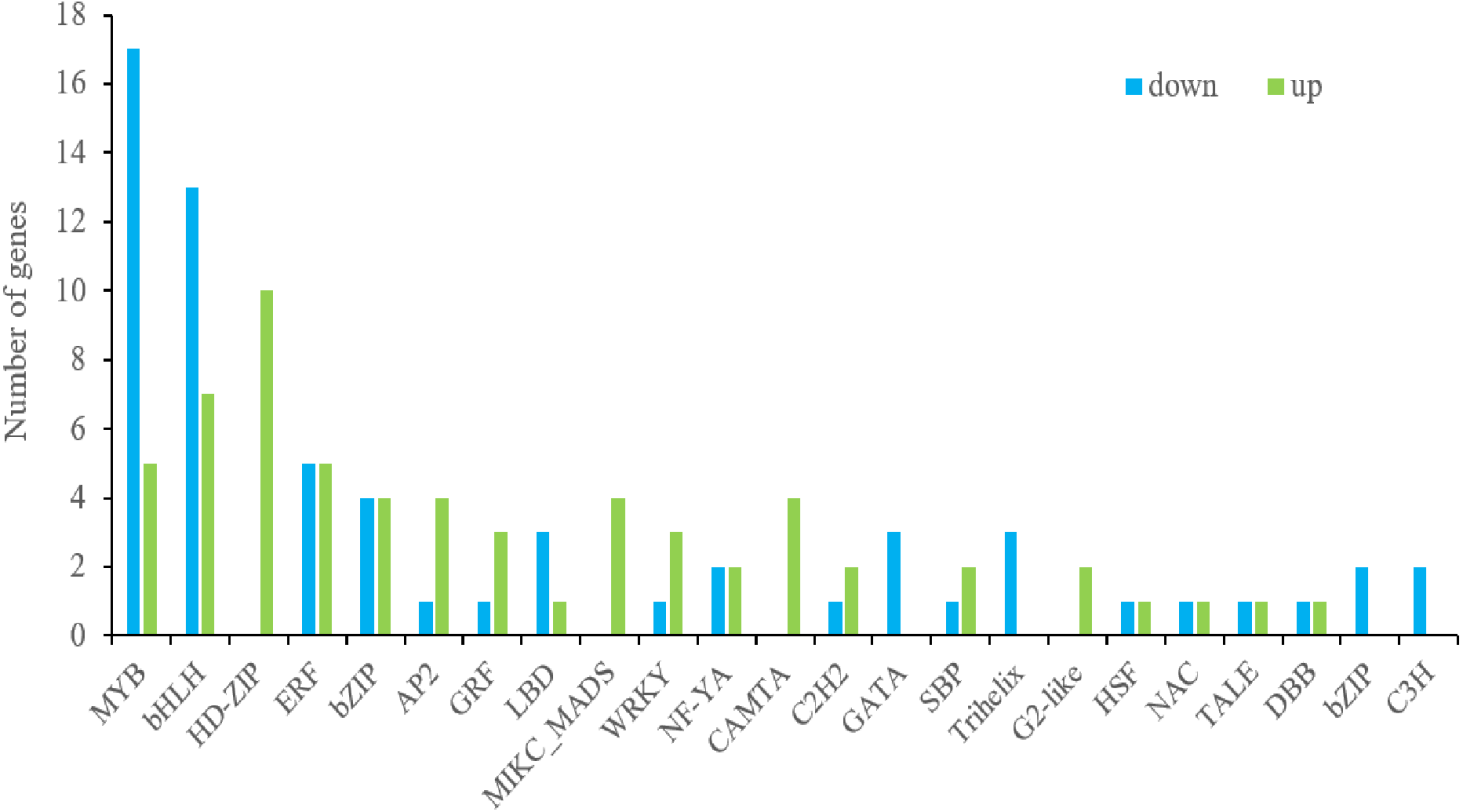
Statistical column diagram showing transcription factors between up- and down-regulated differentially expressed genes (DEGs).

To assess the complex network of signaling pathways in light-induced anthocyanin biosynthesis, we further compared the expression profiles of transcription factors. A total of 133 genes were assigned to the MapMan “transcription factors” bin and more than half were down-regulated (Fig. 6). Members of the GATA, Trihelix, bZIP and C3H families were downregulated and HD-ZIP, MIKC_MADS, CAMTA, G2-like families were upregulated, while other TF families were regulated in both directions. Of these TFs, MYB and bHLH constituted the largest family with 22 and 20 members, followed by the HD-ZIP, ERF, bZIP, AP2, GRF, LBD, MIKC_MADS, WRKY, NF-YA and CAMTA families, with 10, 10, 8, 5, 4, 4, 4, 4, 4, 4 members, respectively. Most of the MYB and bHLH TFs were downregulated, including MYB90, MYB114, EGL3 and TT8, which are key regulators of anthocyanin biosynthesis.

**Figure 7:**
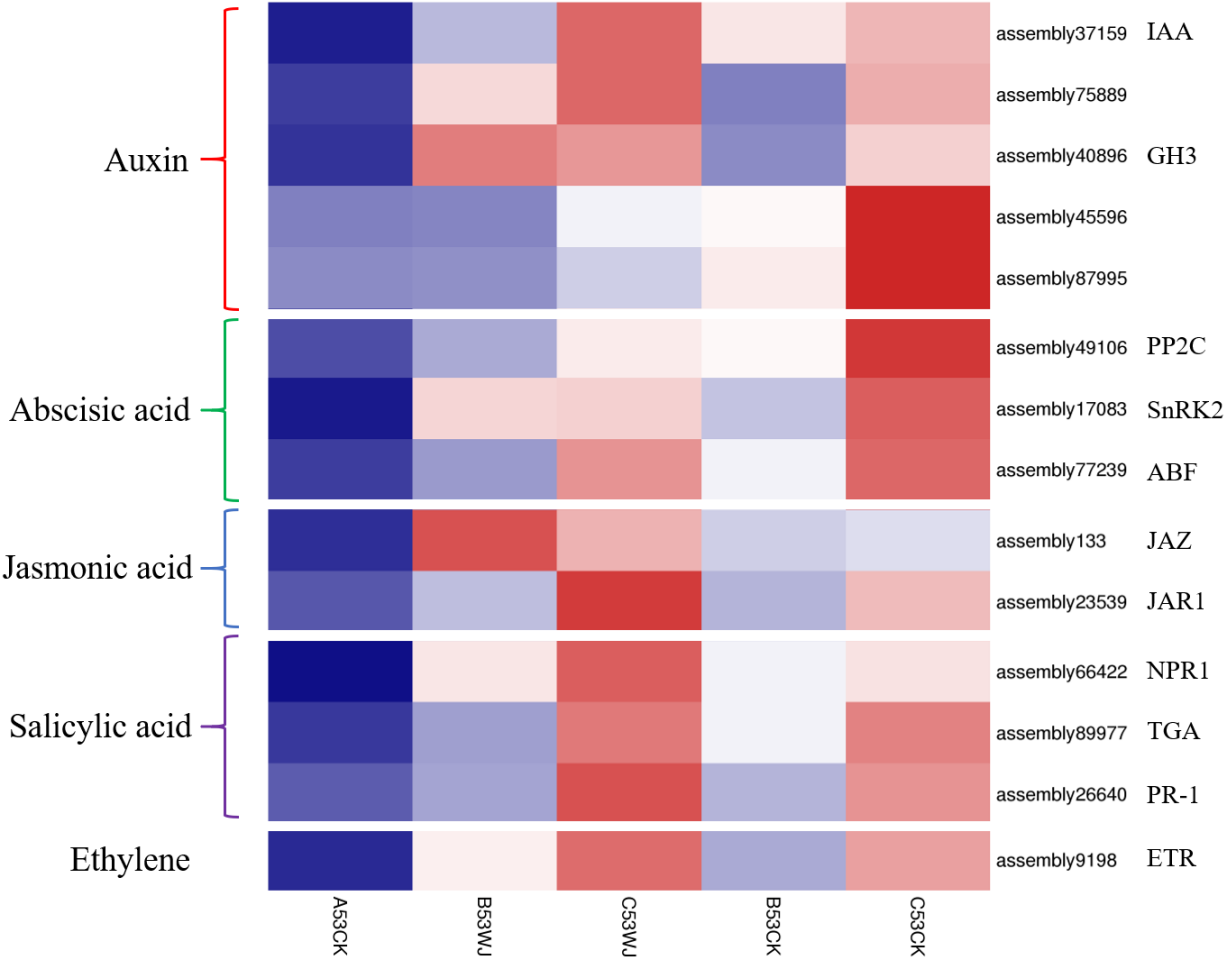
Heat map representation of the expression patterns of DEGs related to the phytohormone biosynthesis and signaling pathways during light-responsive reactions. A53CK, B53WJ, C53WJ, B53CK and C53CK indicate samples treated by head-shading for 0 d, 7 d and 11 d, and normal light for 7 d and 11 d, respectively. The color scale reflected the log-transformed FPKM values.

The interaction partners of these TFs activated as molecular responses are key components of signal transduction pathways that take place during anthocyanin biosynthesis. To investigate the functions of plant hormones in light-induced anthocyanin biosynthesis, the expression patterns of genes involved in plant hormone response as receptors and response factors were assessed by heat map analysis (Fig. 7). The results showed that multiple genes involved in abscisic acid, auxin, salicylic acid, ethylene and jasmonic acid signaling pathway were mostly upregulated in samples treated for 11 d in comparison with those treated for 0 d and 7 d.

In the auxin transduction pathway, the expressions levels of genes encoding auxin influx carrier/auxin-responsive protein IAA (AUX/IAA) (assembly 37159 and assembly 75889) and auxin responsive GH3 gene family (GH3) (assembly 40896) peaked in the C53WJ sample treated by head-shading for 11 d. Meanwhile, the other two GH3 genes (assembly45596 and assembly 87995) were significantly up-regulated in the C53CK sample treated by normal light for 11 d. In the abscisic acid transduction pathway, genes encoding protein phosphatase 2C (PP2C) (assembly 49106), serine/threonine protein kinase SRK2n (SnRK2) genes (assembly17083) and ABRE binding factors (ABF) (assembly 77239) were downregulated under head-shading conditions in comparison with normal light conditions. In the jasmonic acid transduction pathway, the transcriptional level of jasmonate ZIM (JAZ) domain-containing gene (assembly133) was highest in the B53WJ sample treated by headed-shading for 7 d, while gene encoding jasmonic acid resistant 1 (JAR1) (assembly23539) was highly expressed in the C53WJ sample treated by head-shading for 11 d. In the salicylic acid (SA) signaling pathway, SA receptors Non-Expresser of Pathogenesis Related Gene 1 (NPR1) genes (assembly 66422), TGA factor (assembly 89977) and pathogenesis-related 1 (PR-1; assembly 26640) genes were upregulated in the C53WJ sample treated by head-shading for 11 d. These results indicated that gene expression patterns in plant hormone signal transduction pathways showed alterations in broccoli during the shading treatment.

### Different expression patterns of anthocyanin biosynthetic regulatory genes

**Figure 8:**
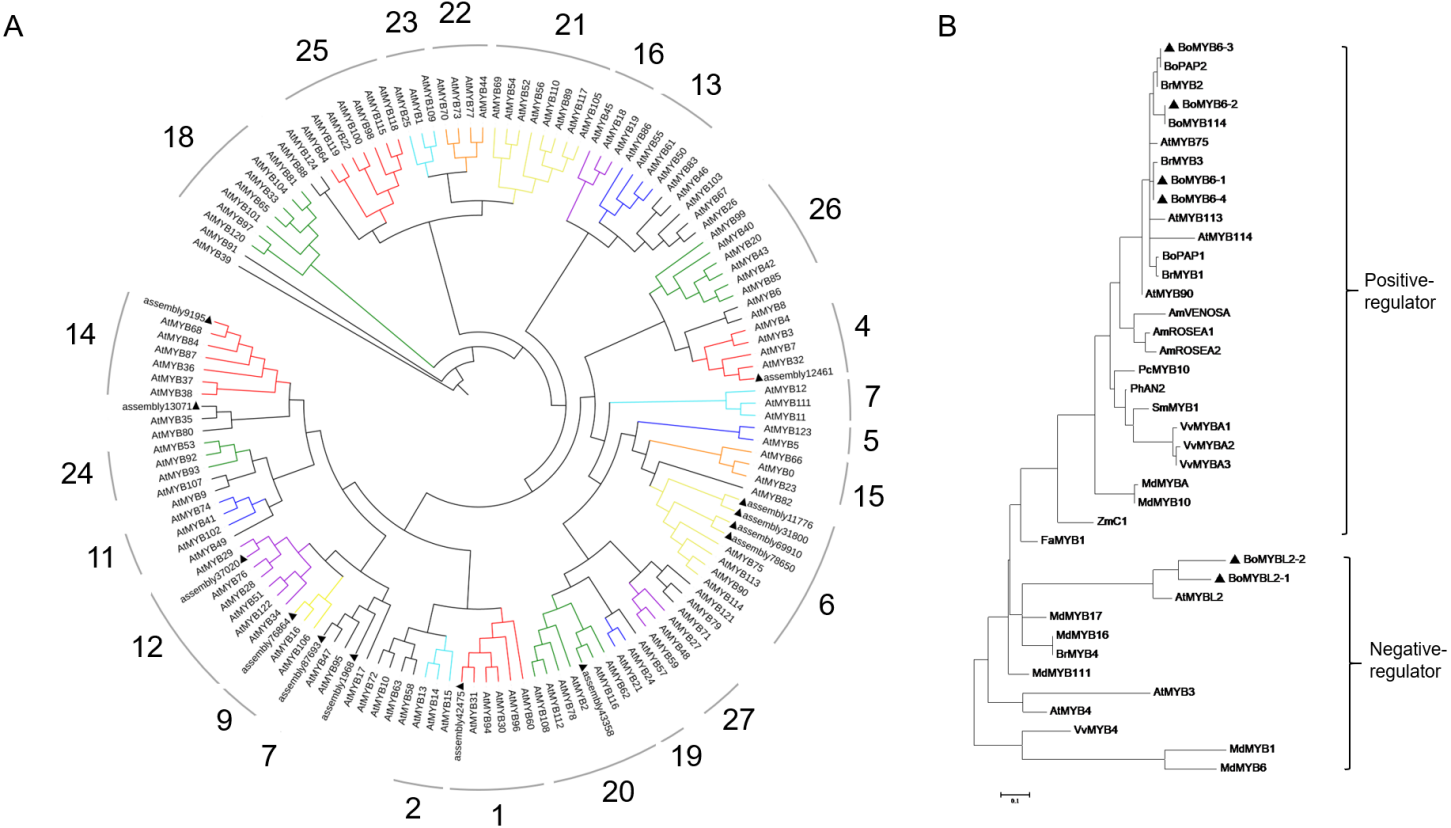
Phylogenetic relationship of BoMYBs with other R2R3-MYBs. The UPGMA method was employed for tree generation using 1,000 bootstrap values with Mega 6.0. Putative modulatory functions of respective R2R3-MYB proteins are shown. (A) Evolutionary relationships of 125 AtMYBs and BoMYBs; (B) Phylogenetic relationships of BoMYBs controlling anthocyanin production and those of other species. Triangles indicate the putatively encoded BoMYB TFs.

To classify and assess the potential functions and evolutionary characteristics of BoMYB proteins in broccoli, a phylogenetic tree was generated for the 125 AtMYBs of Arabidopsis. Thirteen broccoli R2R3-MYBs were clustered together with AtMYBs and grouped into 8 MYB sub-categories (Fig. 8). In the present study, there were one BoMYB in subfamily 1 (S1; assembly 42475), one in S4 (assembly 12461), four in S6 (assembly 69910, assembly 31800, assembly 11776 and assembly 78650), two in S7 (assembly 87693 and assembly 1968), one in S9 (assembly 76864), one in S12 (assembly 37020), one in S14 (assembly 9195), one in S20 (assembly 43358), and one in unknown class (assembly 43358). Then, phylogenetic analysis was carried out to identify probable candidate MYBs involved in anthocyanin regulation in purple broccoli. Notably, S6 molecules were found to modulate flavonoid and/or anthocyanin metabolic pathways. The first S6 group genes, i.e., assembly 69910, assembly 31800, assembly 11776 and assembly 78650, were renamed *BoMYB6-1*, *BoMYB6-2*, *BoMYB6-3* and *BoMYB6-4*, respectively, while assembly 75153 and assembly 75155 were renamed *BoMYBL2-1* and *BoMYB2-2*, respectively. Phylogenetic analysis indicated that *BoMYB6-1*, *BoMYB6-2*, *BoMYB6-3* and *BoMYB6-4* were assigned to the positive-regulator group, while *BoMYBL2-1* and *BoMYB2-2* belonged to the negative-regulator category.

**Figure 9:**
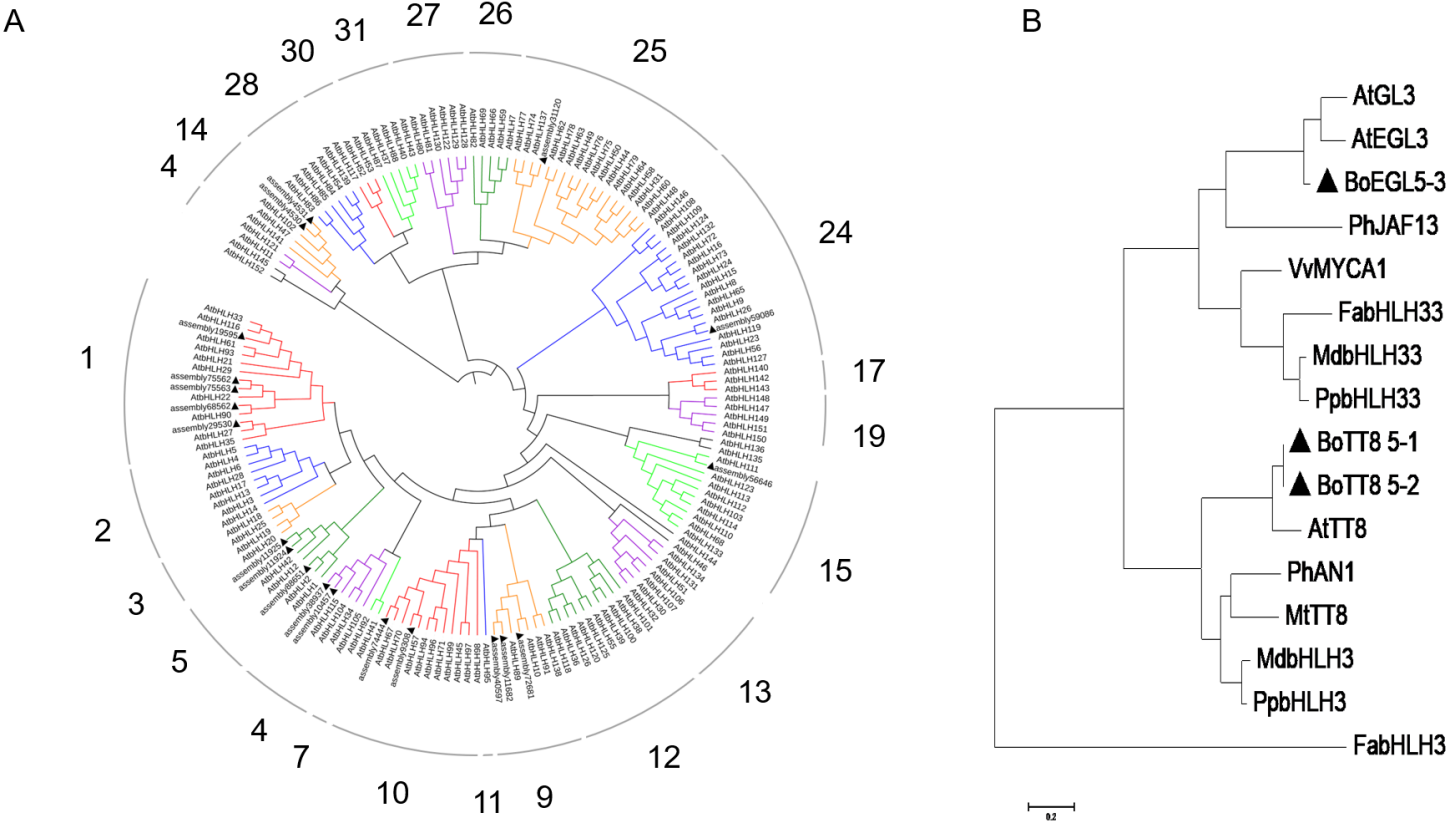
Phylogenetic associations of BobHLH with other bHLHs. The UPGMA method was employed for tree generation using 1,000 bootstrap values with Mega 6.0. Putative modulatory functions of respective BobHLH proteins are shown. (A) Evolutionary relationships of 152 AtbHLHs and BoHLHs; (B) Phylogenetic relationships of BoHLHs controlling anthocyanin production in subfamily 5 and those of other species. Triangles indicate the putatively encoded bHLH TF.

Using the same methods, a total of twenty broccoli BobHLHs and 152 Arabidopsis bHLHs were employed to generate a phylogenetic tree, and divided into eight subfamilies, including 1, 4, 5, 10, 14, 15, 24 and 25. As shown in Fig. 9, subfamily 5 was associated with flavonoid and/or anthocyanin biosynthesis, including three BobHLHs (assembly 11924, assembly 11925 and assembly 88651, renamed *BoTT8_5-1*, *BoTT8_5-2* and *BoEGL5-3*, respectively). Phylogenetic analysis showed BobHLH clustering with Arabidopsis anthocyanin bHLH regulator, *BoTT8_5-1* and *BoTT8_5-2* showed a more distant association with Arabidopsis *AtTT8; BoEGL5-3* had more distant associations with Arabidopsis *AtGL3* and *AtEGL3*. These results suggested that *BoTT8_5-1*, *BoTT8_5-2* and *BoEGL5-3* played roles in regulating anthocyanin synthesis.

### Validation of the expression of genes of the Anthocyanin Biosynthetic Pathway

**Figure 10:**
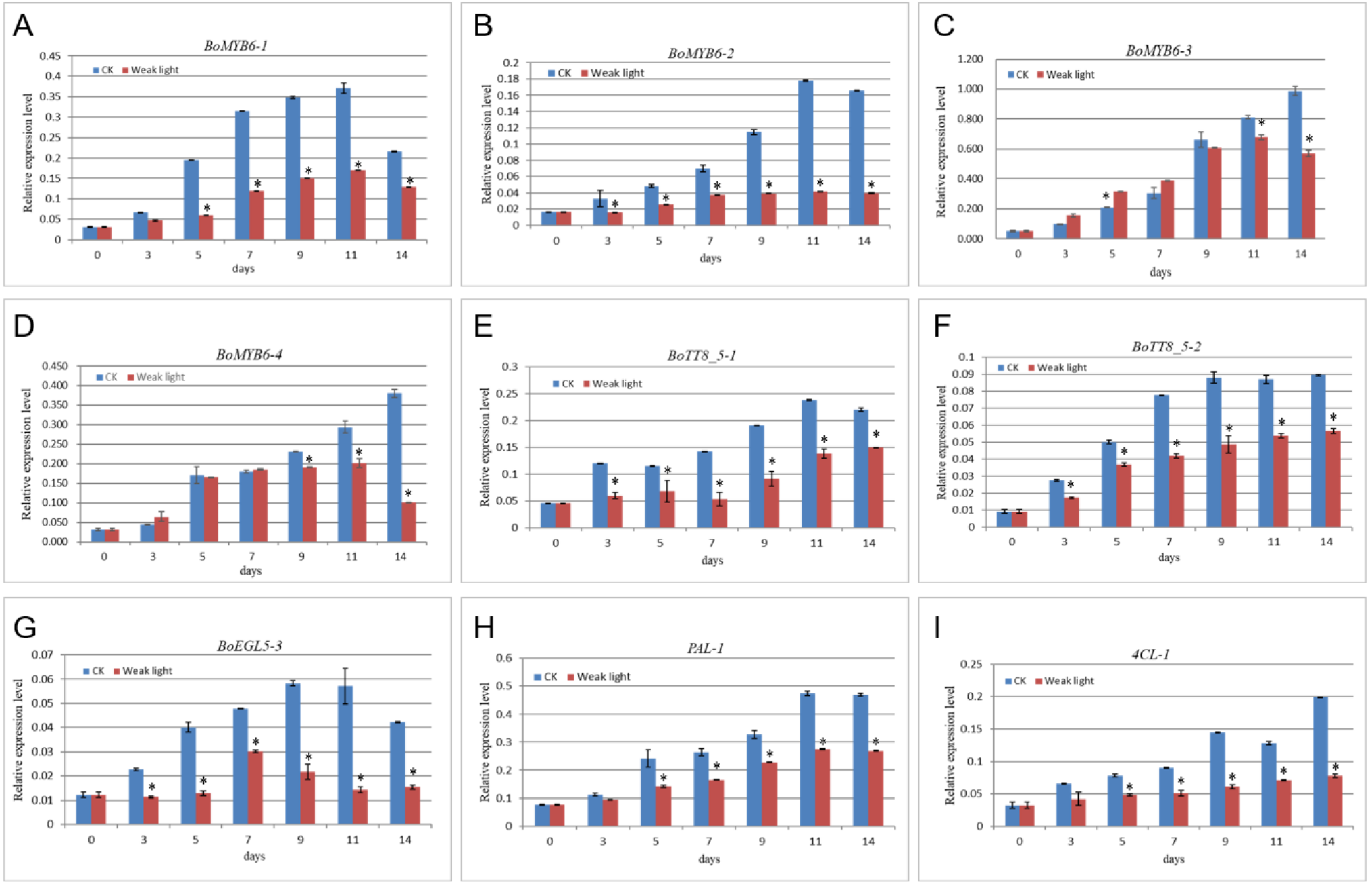
Validation of the expression of genes associated with anthocyanin biosynthesis in broccoli. ^∗^ represents significance at the 0.05 level.

To confirm gene expression data revealed by transcriptomics, seven regulatory genes (*BoMYB6-1*, *BoMYB6-2*, *BoMYB6-3*, *BoMYB6-4*, *BoTT8_5-1*, *BoTT8_5-2* and *BoEGL5-3*) and two structural genes (*PAL1*, *4CL-1*) contributing to anthocyanin production in broccoli underwent amplification from specimens obtained in head’s developmental stages under shading and light conditions, respectively, by RT-qPCR (Fig. 10), with *Actin2* employed as a reference gene. All 9 genes displayed identical expression trends obtained by RNA-seq. Most of the transcription factors assessed also showed identical expression trends as determined by transcriptomics. Moreover, the light and shading groups were significantly different (*P* < 0.05).

## Discussion

### Broccoli head is the key light-response receptor in anthocyanin production

The purple broccoli has abundant flavonoids and other bioactive molecules in addition to glucosinolate-derived isothiocyanates, vitamins and minerals, indicating a great nutritional value for this plant (Moreno et al., 2010). In the present study, delphinidin-3-O-galactoside, delphinidin-3-O-glucoside, cyanidin-3-O-galactoside, cyanidin-3-O-glucoside, malvidin-3-O-galactoside and malvidin-3-O-glucoside were identified in broccoli head, in accordance with previously published data on broccoli sprouts, purple Graffiti cauliflower and red cabbage (Moreno et al., 2010; Chiu et al., 2010; Yuan, Chiu & Li, 2009). Light represents a predominant environmental stimulus controlling plant anthocyanin production (Liu et al., 2018). As shown above, shading reduced anthocyanin production in broccoli. Under light conditions, anthocyanins were produced in a rapid manner (Fig. 1). In this study, the relative contents of total anthocyanins under head-shading were more reduced than those obtained under leaves shading and normal light conditions. This was similar to anthocyanin accumulation in chrysanthemum under head-shading and leaf shading conditions as reported by Hong et al. (2015).

Usually, more focus is placed on fresh broccoli heads which provide economic benefits directly; however, purple broccoli varieties with purple or green leaves are variable in leaf pigmentation. In order to illustrate this mechanism, RNA-seq was performed under head-shading and leaves-shading treatment. Downregulated DEGs, GO and KEGG analyses were carried out. GO analyses showed that, “nucleus”, “response to abscisic acid”, “DNA-binding transcription factor activity”, “response to water deprivation” and “sequence-specific DNA binding” were the major GO terms. Moreover, KEGG analyses showed that, “Starch and sucrose metabolism”, “Plant hormone signal transduction” and “Protein processing in endoplasmic reticulum” were the major public pathway-related database. ABA played positive role in modulating anthocyanin biosynthesis (Carvalho, Carvalho & Duque, 2010). Starch degradation and Sucrose-specific contribute to anthocyanin biosynthesis, while MdSnRK1.1 interacts with MdJAZ18 to induce sucrose-induced anthocyanin and proanthocyanidin biosynthesis in apple (Liu et al., 2017), however IAA might play an crucial role in anthocyanin accumulation regardless of sugar and starch in ornamental kale (Ren et al., 2019). Endoplasmic reticulum likely function in the biosynthesis and transport of anthocyanin pigments (Wagner, 1987). Therefore, the downregulated genes in hormone signaling, starch and sucrose metabolism, endoplasmic reticulum and transcription factors might affect the anthocyanin accumulation under head-shading treatment in broccoli.

Although anthocyanin biosynthesis is well characterized, the associated light-response receptors in broccoli are less clear. Photosynthesis was significantly repressed under both head and leaves-shading conditions, suggesting that shading affects plants during photosynthesis. The DEGs in “photosynthesis”, “peroxisome”, “flavonoid biosynthesis” were significantly repressed by head shading while “amino acid biosynthesis”, “carbon metabolism”, “sulfur metabolism”, “cysteine and methionine metabolism”, and “photosynthesis-antenna proteins” were significantly suppressed by leaves shading via transcriptional regulation. We can infer that under leaves-shading treatment, carbon fixation and carbohydrate production were affected by less light indirectly leading to less anthocyanin contents (Shao et al., 2014). Under head-shading conditions, some photoreceptors and anthocyanin biosynthesis-associated genes downregulated directly leading to decreased anthocyanin contents in response to light. Therefore, the head might be the more critical role as light-response receptor in anthocyanin production.

### Light-induced anthocyanin biosynthesis is mediated by signal transduction pathways in ‘Long Jing’

Plants use many photoreceptors for coordinating responses to environmental light (Ma et al., 2019). When broccoli flowers under head-shading conditions were compared with those under normal light conditions, we found three *CRY3* genes were downregulated, in agreement with the tendency of anthocyanin production in broccoli head flower (Fig. 1B). These finding indicate that CRY3 may be an important photoreceptor in broccoli head flower, with critical functions in regulating anthocyanin production (Opseth et al., 2015). Li et al. (2017) and Li et al. (2018) characterized the eggplant photomorphogenic factors *CRY3* was upregulated by light. In the current study, *HY5* was identified among DEGs during head flower development and its transcription levels quickly rose under light conditions but were reduced under head-shading treatment. In Turnip (*Brassica rapa*), the upregulation of *BrHY5* further induced *BrPAP1* expression to produce more anthocyanins under sunlight (Yang et al., 2017). Shin et al. (2013) has reported that *HY5* induced anthocyanin accumulation by directly binding the promoter of *MYB75/PAP1* transcription factor in *Arabidopsis*. In apple, MdHY5 also bound on the 5′ upstream region of *MdMYBA* in a yeast system (Peng et al., 2013). This suggests *HY5* might represent an inducer of broccoli head flower coloration by binding the promoter of MYB TFs under light conditions (Stracke et al., 2010; Shin et al., 2013; Nguyen et al., 2015).

### Transcription factors are involved in light-induced broccoli coloration

A set of TFs are considered to regulate anthocyanin biosynthesis at the transcriptional level, including R2R3-MYB, bHLH and WD40, as well as members of several other TF families. In this study, 133 transcription factors were differently expressed in response to light which were classified into 23 families (Fig. 6). Among them, *HY5* (Shin et al., 2013), *MYB90* (Bac-Molenaar et al., 2015), *MYB114* (Gonzalez et al., 2008), *MYBL2* (Dubos et al., 2008), *EGL3* (Nemie-Feyissa et al., 2015), *TT8* (Li et al., 2017) and *WRKY26* (Amato et al., 2017), *WRKY70* (Li et al., 2006) were recorded in the study, which were known for their response to anthocyanin biosynthesis. As is well known, HY5 and MYB could triggered expression of light-inducible genes *CHS* after light exposure (Chattopadhyay et al., 1998). In our dataset, the differently expressed R2R3MYB TFs *BoMYB6-1*, *BoMYB6-2*, *BoMYB6-3*, *BoMYB6-4* as well as bHLH TFs *BoTT8_5-1*, *BoTT8_5-2*, *BoEGL5-3*, were categorised into subfamily 6 and 5 respectively, which were found to modulate flavonoid and/or anthocyanin metabolic pathways (Figs. 8 and 9). In ornamental cabbage, R2R3MYB TFs *BoPAP2*, bHLH TFs *BoTT8*, *BoEGL3.1* and *BoMYC1.2* and WDR genes *BoTTG1* were identified as candidates involved in the anthocyanin biosynthesis pathway (Jin et al., 2018). Ectopic expression of the R2R3 MYB TFs *Pr-D* allele in cauliflower induced tissue-specific anthocyanin accumulation (Chiu et al., 2010). In the turnip seedlings, *PAP1* transcript levels simultaneously depend on light spectra and hypocotyl localization, suggesting that MYBs have critical functions in light spectrum- and location-dependent transduction of light signals (Wang et al., 2012). In turnip, *BrTT8*, interacting with *BrPAP1* and *BrTTG1*, plays a positive role in anthocyanin biosynthesis via regulating expression of LBGs (*BrDFR*, *BrANS1*, *BrANS2*, and *BrUFGT*) in response to light (Yang et al., 2017). In addition, many other differently expressed TFs response to light were detected in our datasets, especially some MYB genes, MADS-box and NAC, suggesting their potential roles in regulation of anthocyanin accumulation (Jaakola, Poole & Jones, 2010; Wang et al., 2018). As a negative regulator, loss of *BoMYBL2-1* expression led to the establishment of purple color in cabbages (Song et al., 2018). Moreover, *MYBL2* expression was inhibited by high light-induced stress, which triggered strong accumulation of anthocyanins in *Arabidopsis* (Dubos et al., 2008). At the present study, we screened numerous differently expressed TFs, including 10 *HD-ZIPs*, 10 *ERFs*, 8 *bZIPs*, 5 *AP2*, 4 *WRKYs*, 4 *GRFs*, 4 *LBDs*, 4 *MIKC_MADS*, 4 *NF-YAs* and 4 *CAMTAs* families. Also, further studies are needed to ascertain their roles in anthocyanin biosynthesis in response to light in broccoli.

### Plant hormones are involved in light-induced broccoli coloration

Previous studies have shown that phytohormones controlling anthocyanin accumulation can be affected by light (Loreti et al., 2008; Zhang et al., 2016a; Zhang et al., 2016b). Endogenous application of auxins can inhibit the expression of anthocyanin-related genes (Deikman & Hammer, 1995). In this study, the expressions levels of IAAs and one GH3 genes were higher in the C53WJ sample treated by head-shading for 11 d. However, two GH3s were upregulated in the C53CK sample treated by normal light for 11 d. These distinct expression patterns indicate that auxin signaling has different functions in the regulation of anthocyanin biosynthesis in broccoli through various transduction pathways. In purple ornamental cabbage, Jin et al. (2019) suggested that ABA might increase the intensity of purple pigmentation of the inner leaves. Carvalho, Carvalho & Duque (2010) provided evidence that ABA plays a positive role in modulating anthocyanin biosynthesis in hormone mutants after exogenous application (Carvalho, Carvalho & Duque, 2010). We observed significant upregulation of genes encoding ABA-responsive elements, such as PP2C, SnRK2 and ABF in the normal light treatment (Fig. 7). Thus, we speculate that these ABA signaling factors might promote the expression of anthocyanin-related genes. Wang et al. (2019) indicated that ethylene acts as a negative regulator in red light-regulated anthocyanin biosynthesis in cabbage. Ethylene suppresses the anthocyanin biosynthesis via binding to ETRs in *Arabidopsis* (Jeong et al., 2010) and peel (Ma et al., 2019). Similarly, we observed that the expression levels of ETR were higher under the head-shading treatments. Previous studies have shown ethylene treatment significantly lowers anthocyanin accumulation, while SA alleviates these effects in canola plants (*Brassica napus L*.) (Tirani, Nasibi & Kalantari, 2013). ABA, JA and SA pre-treatments could increase anthocyanin accumulation in turnip (*Brassica rapa ssp. rapa*) and *Brassica juncea L*. (Thiruvengadam et al., 2016; Sharma et al., 2018). Horváth et al. (2007) reported exogenously applied SA increases the accumulation of anthocyanin in UV-B exposed *T. aestivum*. Endogenous application of jasmonate can also increase the production of anthocyanin (Memelink). However, we found that all genes encoding *JAZ*, *JAR1* and *NPR1* in jasmonic acid signalling pathway as well as TGA and PR-1 in the salicylic acid signaling pathway were upregulated under head-shading treatment, which implies that SA and JA play negative roles in light-induced anthocyanin accumulation in broccoli. In general, some form of hormonal cross-talk may be present in pigment accumulation of broccoli flower head.

## Conclusions

Overall, comprehensive gene expression analysis was performed for exploring the mechanisms underlying light-associated anthocyanin accumulation in broccoli, and the following regulatory sequence was proposed. Under light conditions, the expression levels of *CRY3* and *HY5* contributing to light signal perception and transduction are closely associated with anthocyanin production in broccoli head flower. *HY5* activates the downstream MYBs related to anthocyanin biosynthesis. Strikingly, the above results revealed that *BoMYB6-1*, *BoMYB6-2*, *BoMYB6-3*, *BoMYB6-4*, *BoMYBL2-1*, *BoMYBL2-2*, *BoTT8_5-1*, *BoTT8_5-2* and *BoEGL5-3* affect anthocyanin production and regulate structural genes such as *PAL*, *C4H*, *4CL*, *CHS*, *CHI*, *F3H* and *DFR*. In addition, HD-ZIP, ERF, bZIP, AP2, GRF, LBD, MIKC_MADS, WRKY, NF-YA, CAMTA transcription factors and abscisic acid, auxin, salicylic acid, ethylene, jasmonic acid signaling pathway involved in the anthocyanin biosynthesis in response to light. This study provides novel insights into the functions of these genes in regulating light-associated anthocyanin production.

##  Supplemental Information

10.7717/peerj.8870/supp-1 Figure S1 GO enrichment analysis for down-regulated DEGs in B53WJ-vs-A53WJ

10.7717/peerj.8870/supp-2 Figure S2 GO enrichment analysis for down-regulated DEGs in A53WJ-vs-B53CK

10.7717/peerj.8870/supp-3 Figure S3 GO enrichment analysis for down-regulated DEGs in B53WJ-vs-B53CK

10.7717/peerj.8870/supp-4 Figure S4 Patterns of gene expressions across three time points under head-shaded condition

10.7717/peerj.8870/supp-5 Figure S5 Patterns of gene expressions across three time points under normal light condition

10.7717/peerj.8870/supp-6 Table S1 Raw data for anthocyanin content

10.7717/peerj.8870/supp-7 Table S2 GO enrichment analysis for down-regulated DEGs in B53WJ-vs-A53WJ

10.7717/peerj.8870/supp-8 Table S3 GO enrichment analysis for down-regulated DEGs in A53WJ-vs-B53CK

10.7717/peerj.8870/supp-9 Table S4 GO enrichment analysis for down-regulated DEGs in B53WJ-vs-B53CK

10.7717/peerj.8870/supp-10 Table S5 Reads Mapping stats

10.7717/peerj.8870/supp-11 Table S6 de novo assembly and annotation

10.7717/peerj.8870/supp-12 Table S7 Assemble unigenes FPKM value

10.7717/peerj.8870/supp-13 Table S8 Specially expressed DEGs in cluster 8 under head shading condition

10.7717/peerj.8870/supp-14 Table S9 Specially expressed DEGs in cluster 8 under normal light condition

10.7717/peerj.8870/supp-15 Table S10 Co-expressed DEGs in cluster 8

## Additional Information and Declarations

### Data Availability

The following information was supplied regarding data availability: Data is available in Table S1 and at NCBI SRA: PRJNA560282.
