# Supplementary figures and images for: Transcriptomic profiling of purple broccoli reveals light-induced anthocyanin biosynthetic signaling and structural genes
Source: PeerJ. 2020 May 5;8:e8870. doi: 10.7717/peerj.8870 (PMC7207213; doi:10.7717/peerj.8870)

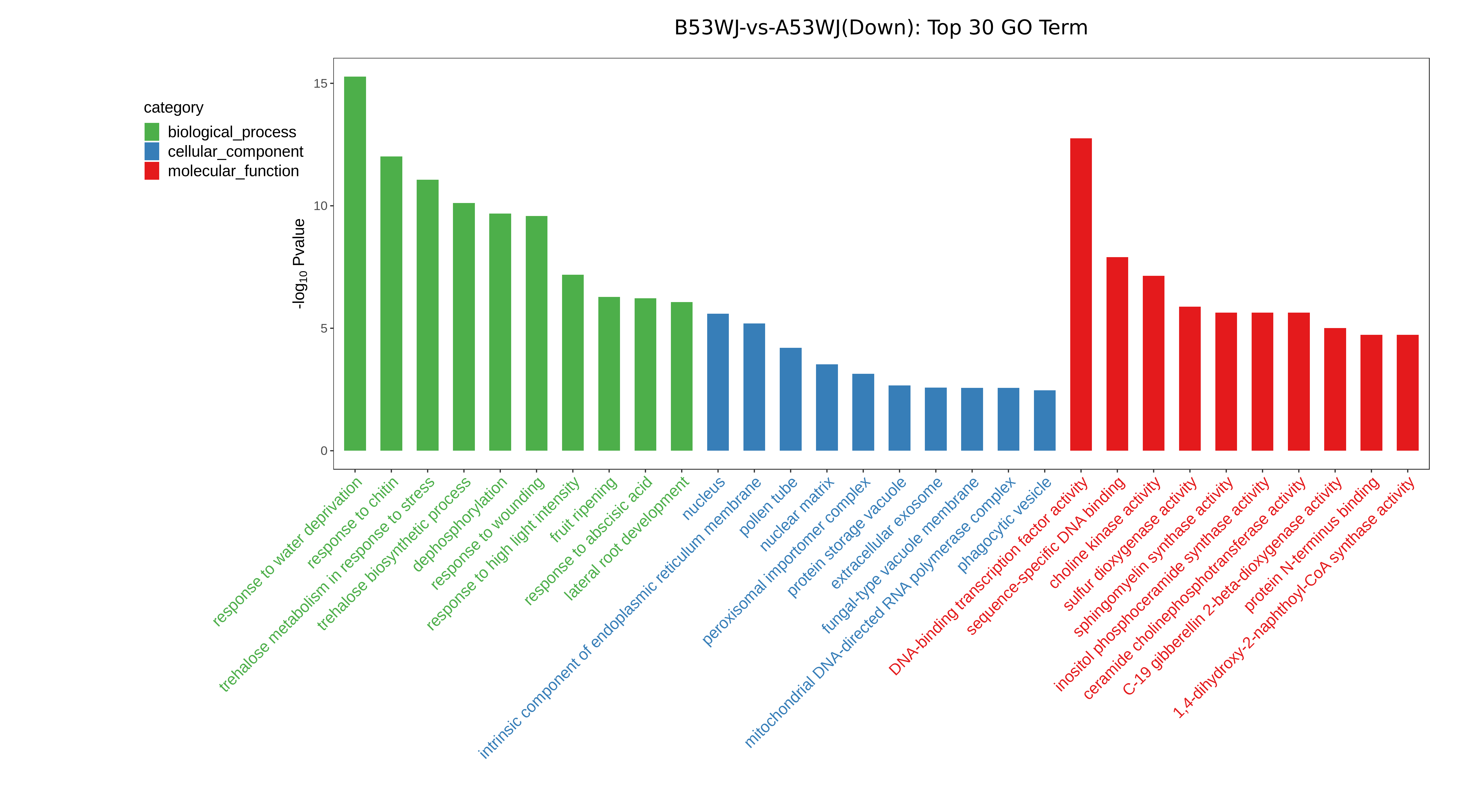

Supplement: Figure S1 [file peerj-08-8870-s001.png]

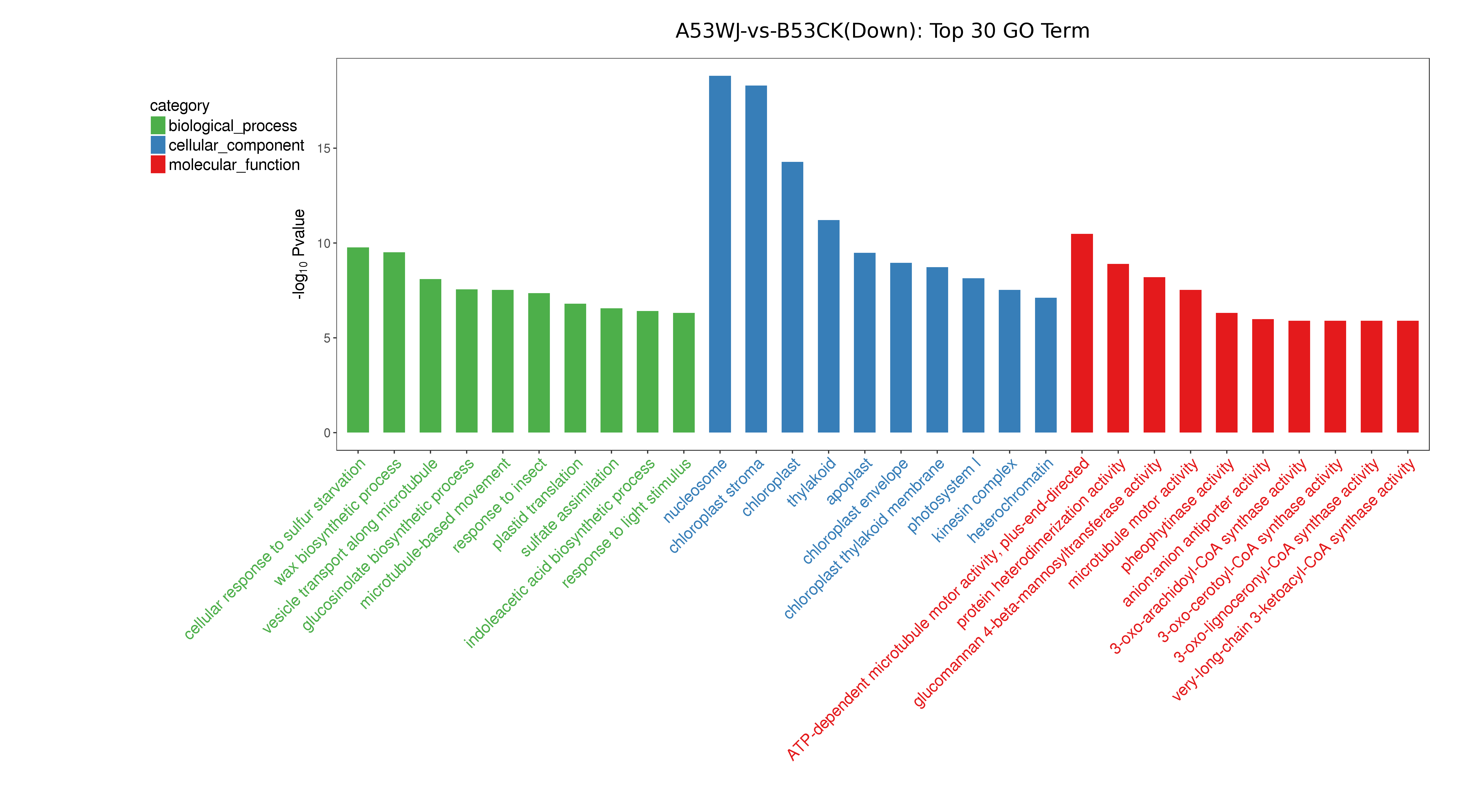

Supplement: Figure S2 [file peerj-08-8870-s002.png]

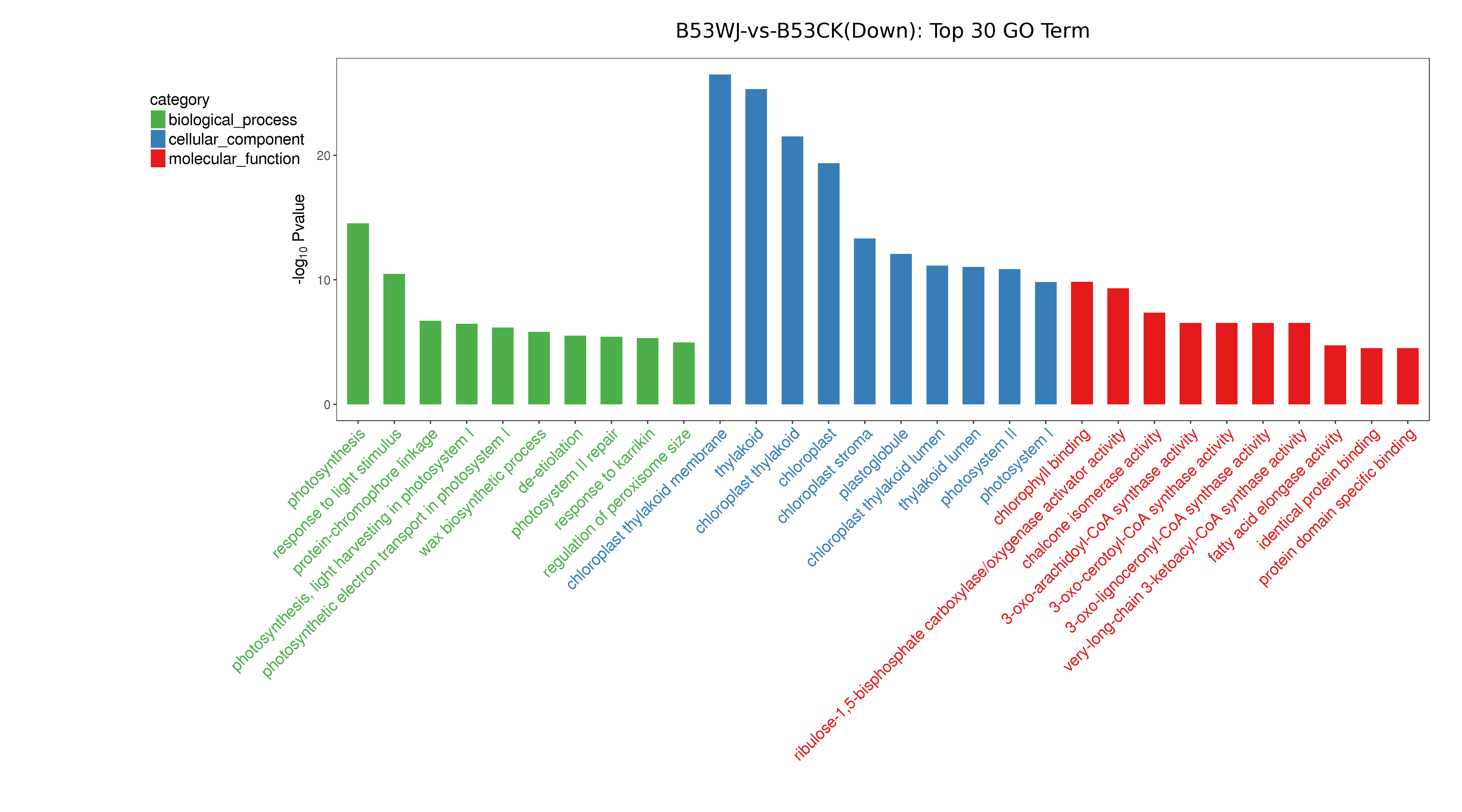

Supplement: Figure S3 [file peerj-08-8870-s003.png]

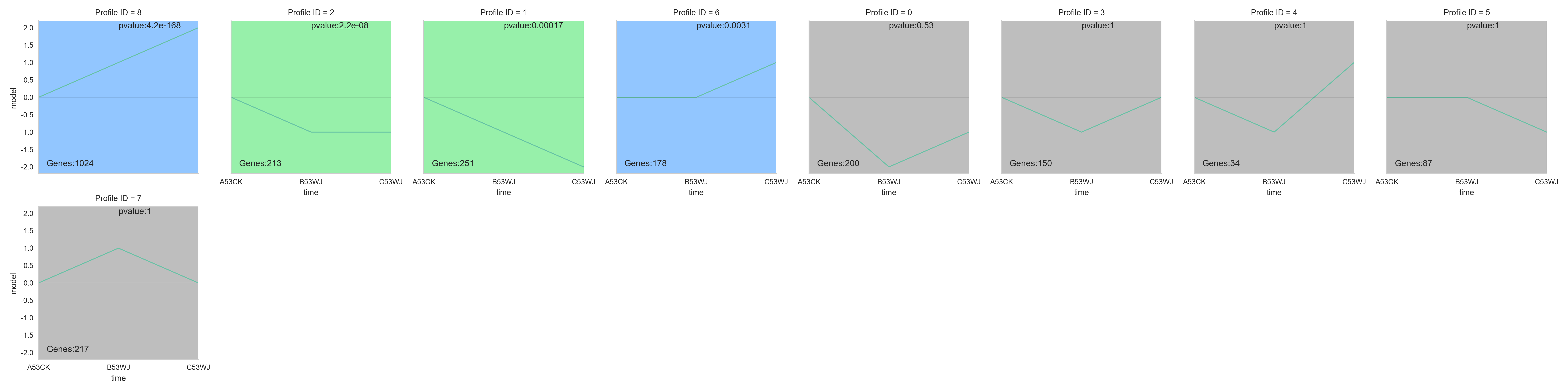

Supplement: Figure S4 [file peerj-08-8870-s004.png]

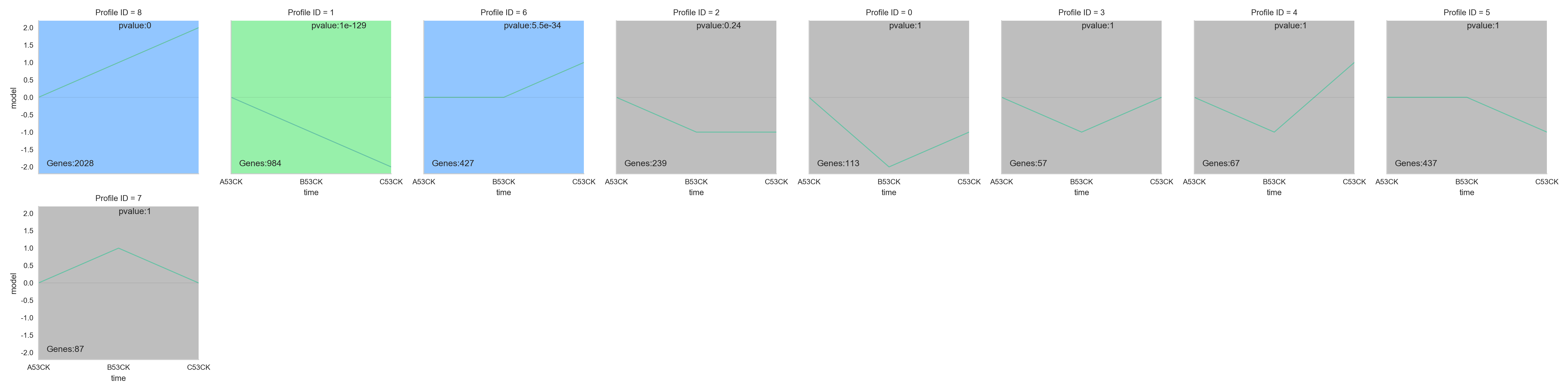

Supplement: Figure S5 [file peerj-08-8870-s005.png]
